# Mr. Vaughan Henry Alexander Holberton

**Published:** 1910-05-07

**Authors:** 


### OBITUARY.

Mr. Vaughan Henry Alexander Holberton has died in South Devon, aged seventy-seven. He was a member of the Royal College of Surgeons and a licentiate of the Society of Apothecaries.

